# Central Arterial Dynamic Evaluation from Peripheral Blood Pressure Waveforms Using CycleGAN: An In Silico Approach

**DOI:** 10.3390/s23031559

**Published:** 2023-02-01

**Authors:** Nicolas Aguirre, Leandro J. Cymberknop, Edith Grall-Maës, Eugenia Ipar, Ricardo L. Armentano

**Affiliations:** 1 GIBIO, Facultad Regional Buenos Aires, Universidad Tecnológica Nacional, Buenos Aires C1179AAQ, Argentina; 2 LIST3N, Université de Technologie de Troyes, 10004 Troyes, France

**Keywords:** arterial stiffness, deep learning, arterial pressure waveform

## Abstract

Arterial stiffness is a major condition related to many cardiovascular diseases. Traditional approaches in the assessment of arterial stiffness supported by machine learning techniques are limited to the pulse wave velocity (PWV) estimation based on pressure signals from the peripheral arteries. Nevertheless, arterial stiffness can be assessed based on the pressure–strain relationship by analyzing its hysteresis loop. In this work, the capacity of deep learning models based on generative adversarial networks (GANs) to transfer pressure signals from the peripheral arterial region to pressure and area signals located in the central arterial region is explored. The studied signals are from a public and validated virtual database. Compared to other works in which the assessment of arterial stiffness was performed via PWV, in the present work the pressure–strain hysteresis loop is reconstructed and evaluated in terms of classical machine learning metrics and clinical parameters. Least-square GAN (LSGAN) and Wasserstein GAN with gradient penalty (WGAN-GP) adversarial losses are compared, yielding better results with LSGAN. LSGAN mean ± standard deviation of error for pressure and area pulse waveforms are 0.8 ± 0.4 mmHg and 0.1 ± 0.1 cm^2^, respectively. Regarding the pressure–strain elastic modulus, it is achieved a mean absolute percentage error of 6.5 ± 5.1%. GAN-based deep learning models can recover the pressure–strain loop of central arteries while observing pressure signals from peripheral arteries.

## 1. Introduction

### 1.1. Arterial Stiffness

Cardiovascular (CV) disease remains the leading cause of death around the world [1]. Early detection is currently one of the main strategies to mitigate occurrence and damage caused by CV disease events. In this sense, increased arterial stiffness is considered a major CV risk factor, jointly with aging, hypertension, smoking, and diabetes, among others, due to its impact on the viscoelastic properties of the arterial wall (mainly in large arteries such as the aortic, carotid, and femoral) [2]. While the elastic function of vascular mechanics is performed by collagen and elastin fibers, the viscous behavior is related to the vascular smooth muscle cells [3]. The viscoelastic dynamics can be addressed through the pressure–strain relationship, in which the delay of the arterial deformation against the pressure produces a hysteresis loop [4]. From a biomechanical perspective, the pressure-diameter (P-D) relationship provides valuable information in terms of the elastic and viscous properties of the arterial wall. In particular, the elastic module (E) is usually obtained from the diastolic phase, whereas the viscous module is estimated from the P-D loop enclosed area [4]. Unfortunately, in vivo measurements of aortic dynamics along this pathway are difficult to obtain due to their invasiveness and the requirement for specialized equipment and trained operators.

### 1.2. Arterial Stiffness and Machine Learning

In recent years, advances in machine learning (ML) have offered different models and algorithms that can be adapted to CV healthcare. In this context, ML model tasks are divided into parameter estimation and subject risk stratification. For instance, systolic blood pressure (SBP) and diastolic blood pressure (DBP) value estimation constitutes a typical approach of the use of both classic ML and deep learning models. In certain cases, classic ML models such as support vector machine (SVM) and fully-connected neural network (NN) implement a feature extraction step in their pipeline. On the contrary, deep learning models have the capacity to generate their own features, meaning that the feature extraction step is not required. Nevertheless, deep learning models require a greater amount of samples to produce better generalization compared to classic ML models. This condition is probably the reason why there seems to be a gap in the use of in vivo datasets jointly with deep learning models. According to the literature, researchers in the CV signal field have focused on a feature-based approach with classic ML, using peripheral blood pressure (BP) or photoplethysmography (PPG) signals rather than deep learning techniques. In Alty et al. [5], several PPG features were extracted and used as the input of a SVM model in order to estimate PWV, a surrogate of regional arterial stiffness. Similarly, in Tavallali et al. [6], features were extracted from the uncalibrated carotid pulse wave (PW) and clinical information was used to train a NN and estimate PWV. In particular, and focusing on the same objective, in Jin et al. [7] both classic ML and deep learning models were compared using in vivo radial PWs.

### 1.3. Virtual Databases in Research

Deep learning models are usually trained using synthetic datasets [8], as the acquisition of real samples, even when possible, turns to out be difficult, expensive, and/or time-consuming. In this context, 1D modeling of arterial PWs, such as that specified in Charlton et al. [9] and Xiao et al. [10], allows the limitations of deep learning samples to be overcome. Furthermore, different profiles or “virtual subjects” are generated by modifying the more representative CV properties, allowing consolidation of a useful database. These simulations can be used then to (1) complement clinical studies [11]; (2) examine methods [12]; or (3) explore the capacity of ML models to explain the relationship between PWs and the underlying CV parameters. In keeping with this approach, Willemet et al. [11] proposed that the occurrence of reflected waves in the late diastole phase affects the foot-to-foot PWV algorithm, causing inaccuracies in PWV estimation. With respect to point (2), Alastruey [12] compared methods for estimating PWV and concluded that those relying on P-D loops were closer to the theoretical values. Finally, regarding ML capacity, several works have proposed estimating CV parameters using a features-based approaches, generally with peripheral arterial information. Bikia et al. [13] used classic ML models to estimate aortic systolic BP, cardiac output, and end-systolic elastance from cuff SBP and DBP, heart rate, and PWV. Ipar et al. [14] investigated a similar approach, extracting features such as systemic vascular resistance from radial and carotid signals. Xiao et al. [15] estimated the stroke volume based on features using only radial signals. Jin et al. [7] examined different signal-to-noise ratios and their impact in terms of percentage error by estimating the PWV from radial pressure PW.

By virtue of the above, it is clear that the research approaches in the literature are consistent with the use of pressure signals from peripheral arteries in estimating parameters related to the properties of the central arteries. Nevertheless, to the best of our knowledge, aortic dynamics have not been addressed in terms of the P-D relationship in a way that is non-invasive using deep learning approaches. This rest of this work is structured as follows. In Section 2, the information obtained from public databases is detailed along with the framework for P-D loop evaluation, the deep learning model, the metrics used to evaluate the results, and the definitions of the different hyperparameter settings used in the experiments. In Section 3, the experimental results of the trained models are reported. In Section 4, the results are discussed and similarities and differences in relation to other works are pointed out. Lastly, in Section 5, a brief conclusion of the present work is summarized.

## 2. Methodology

### 2.1. Dataset

**Figure 1:**
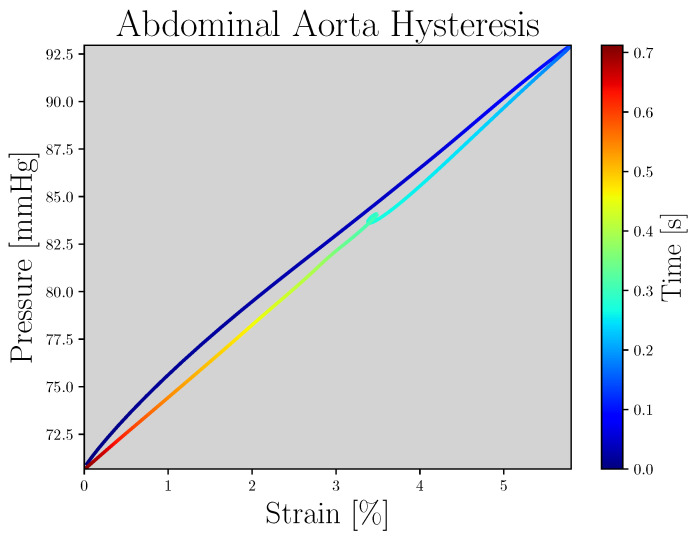
Abdominal aorta pressure–strain hysteresis loop; the color of the line refers to the time.

The dataset used in this work corresponds to the one in Charlton et al. [9]. It is composed of 3837 simulated PWs with different cardiac, vascular, arterial, vascular bed, and blood properties. The simulated PWs’ hemodynamic characteristics exhibit tendencies that are in accordance with those in the literature in terms of both values and morphology [9]. The magnitudes provided by this dataset are pressure (mmHg), luminal area (m^2^) and flow velocity (m/s), corresponding to different sites of the arterial tree. Furthermore, because the arterial wall viscosity property is considered in the database [9], the hysteresis phenomenon between pressure and strain can be evaluated, as shown in Figure 1.

**Figure 2:**
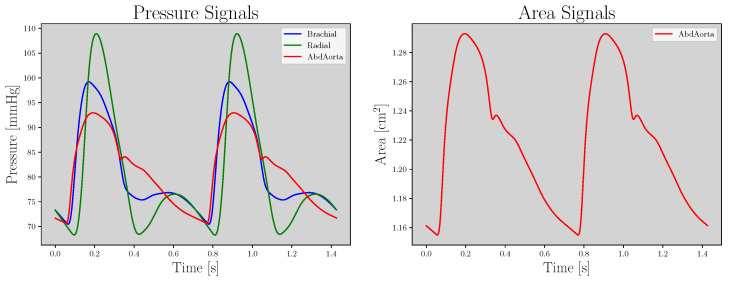
**Left**: Brachial, radial and abdominal aorta pressure signals (blue, green, and red lines, respectively). **Right**: Abdominal aorta area signal.

For the present study, the pressure from the radial and brachial artery (P_Bra-Rad_) signals correspond to the *X* domain (the blue and green lines in Figure 2) and the aortic abdominal pressure and area (P-A_Abdo_) signals correspond to the *Y* domain (the red lines in Figure 2). Although PWs are noiseless, a pre-processing stage is performed to prepare the model’s input. First, the area magnitude is converted to cm^2^. Second, because PWs have different durations, the time-window is fixed to the maximum PW duration across the database; shorter pulses are repeated until time-windows are fulfilled, considering a stationary situation. Third, a MinMax [−1, 1] normalization is performed for both pressure and area magnitude. Finally, PWs are resampled from 500 Hz to 256 Hz, which is done for two reasons: (1) to reduce computational cost, and (2) because 256 Hz is a common sampling frequency used in commercial devices (SphygmoCor®XCEL, AtCor Medical, Sydney, Australia).

### 2.2. Pressure–Strain Elastic Modulus

From a biomechanical point of view, arterial stiffness is defined in terms of E:

(1) E=dσ/dε

where ε refers to the arterial strain and σ to the axial stress, defined as

(2) σ=P·R/h

where *h* is the h, *P* refers to the inner BP, and *R* is the arterial radius. Nevertheless, because *h* is not directly defined in the dataset, a simplification of Equation (1) that only regards *P* and ϵ, called E_P-ε_ [16], is considered as the measurement of arterial stiffness:

(3) $$E_{\mathrm{P-\varepsilon}}=dP/d\varepsilon$$

As detailed in the next section, the proposed model estimates the magnitudes of both pressure and area. Then, in Section 2.4, after the P-D loops are constituted from estimations, E_P-ε_ is observed as an evaluation parameter.

### 2.3. CycleGAN Model

GANs are a type of generative deep learning models composed of a discriminator (*D*) and a generator (*G*). The generator tries to generate samples similar to a given domain in such a way that the discriminator cannot differentiate between real and fake samples. On the contrary, the discriminator tries to recognize real samples from created by the generator. GANs are trained by defining the *Adversarial Loss* ($L_{GAN}$), which is a function that measures the distance between two probabilistic distributions.

The CycleGAN [17] architecture has been proposed to learn mapping functions between two domains, *X* and *Y*, using GANs. Although it was originally proposed for unpaired image-to-image translation, in this work the *X* and *Y* samples are paired, as mentioned before, and correspond to the P_Bra-Rad_ and P-A_Abdo_ signals, respectively.

#### 2.3.1. General Architecture

**Figure 3:**
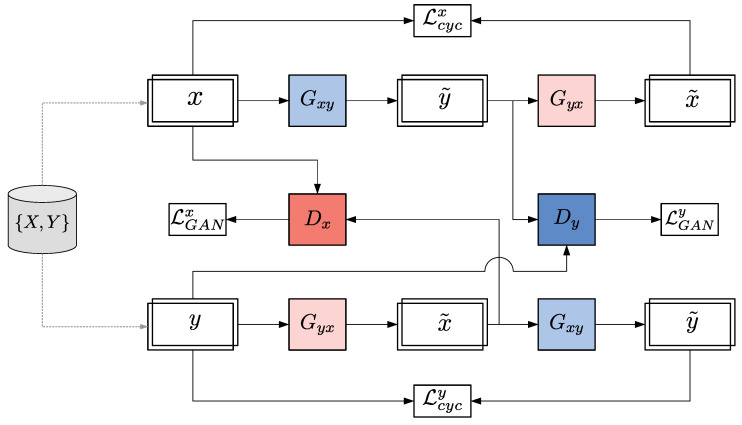
CycleGAN architecture and its training connection flow.

As shown in Figure 3, the mapping functions are $G_{xy}:X\to Y$ and $G_{yx}:Y\to X$, given training samples $x_{i}\in X$ and $y_{i}\in Y$. Two adversarial discriminators $D_{x}$ and $D_{y}$ are incorporated, where the goal of $D_{x}$ is to discriminate signals from *X* and $G_{yx}\left(y\right)$. Similarly, $D_{y}$ aims to distinguish signals from *Y* and $G_{xy}\left(x\right)$. In addition to $L_{GAN}$, a *Cycle-Consistency Loss* ($L_{cyc}$) function is added to force learnable mapping functions to recover their originals inputs.

The full objective function contains two terms [17],

(4) $$\begin{array}{cc} L(G_{xy},G_{yx},D_{x},D_{y})\; & =L_{GAN}\left(G_{xy},G_{yx},D_{x},D_{y},X,Y\right)\\ \; & +\lambda_{cyc}L_{cyc}\left(G_{xy},G_{yx},X,Y\right) \end{array}$$

we expand $L_{GAN}\left(G_{xy},G_{yx},D_{x},D_{y},X,Y\right)$ and $L_{cyc}\left(G_{xy},G_{yx},X,Y\right)$ in detail in Section 2.3.3; moreover, $\lambda_{cyc}$ is a scalar that helps to scale the losses.

#### 2.3.2. Architecture of Generators and Discriminators

**Figure 4:**
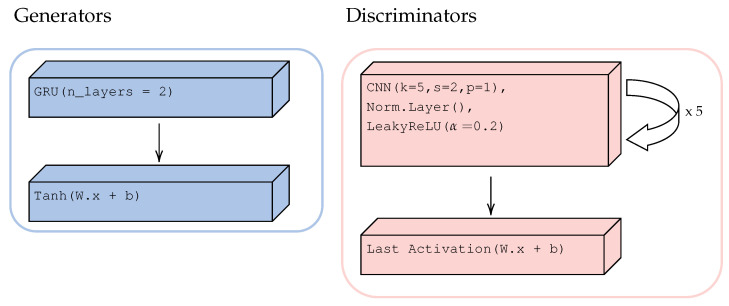
Generator and discriminator components.

In the present work, the generators $G_{xy}$ and $G_{yx}$ are two stacked gated recurrent unit (GRU) layers followed by a linear projection with two out features and ending with a tanh() activation function. Discriminators $D_{x}$ and $D_{y}$ are five stacked layers of convolutional neural networks (CNNs) (kernel, stride, and padding having sizes of 5, 2, and 1, respectively) with a normalization layer and LeakyReLU (α= 0.2) activation function in each layer. At each layer of discriminator, the number of output features is doubled; finally, one last layer of linear projection with one output feature ending with an activation function is added. Depending on the selected $L_{GAN}$, the normalization and last activation layers change; details are provided in Section 2.3.4. Figure 4 shows the structures of *G* and *D*.

#### 2.3.3. Loss Functions

First, considering Figure 3 and the first term in Equation (4), $L_{GAN}$ can be decomposed as follows [17]:

(5) $$L_{GAN}\left(G_{xy},G_{yx},D_{x},D_{y},X,Y\right)=L_{GAN}^{x}\left(G_{yx},D_{x},Y,X\right)+L_{GAN}^{y}\left(G_{xy},D_{y},X,Y\right)$$

To measure $L_{GAN}$, two different metrics have been implemented:

- LSGAN [18]
- WGAN-GP [19]

where LSGAN and WGAN-GP measure the Pearson $\chi^{2}$ divergence and the Wasserstein distance, respectively. For the sake of simplicity, only the case where $G_{xy}:X\to Y$ (the second term in Equation (5)) is written, because $G_{yx}:Y\to X$ is defined analogously by replacing the *X* with the *Y* domain and vice versa. The objective for $L_{GAN}\left(G_{xy},D_{y},X,Y\right)$ considering LSGAN is defined as follows:

(6) $$\begin{array}{cc} \min_{D_{y}}L_{GAN}^{y}\left(G_{xy},D_{y},X,Y\right) & =\frac{1}{2}\underset{y∼Y}{E}\left[{\left(D_{y}\left(y\right)-1\right)}^{2}\right]+\frac{1}{2}\underset{\tilde{y}∼\tilde{Y}}{E}\left[{\left(D_{y}\left(\tilde{y}\right)\right)}^{2}\right]\\ \min_{G_{xy}}L_{GAN}^{y}\left(G_{xy},D_{y},X,Y\right) & =\frac{1}{2}\underset{\tilde{y}∼\tilde{Y}}{E}\left[{\left(D_{y}\left(\tilde{y}\right)-1\right)}^{2}\right] \end{array}$$

where $\tilde{y}=G_{xy}\left(x\right)$ and $\tilde{Y}$ refers to the estimated distribution of *Y*.

When WGAN-GP is considered, the objective for $L_{GAN}^{y}\left(G_{xy},D_{y},X,Y\right)$ is as follows [18]:

(7) $$\begin{array}{cc} \min_{G_{xy}}\min_{D_{y}}L_{GAN}^{y}\left(G_{xy},D_{y},X,Y\right) & =\underset{\tilde{y}∼\tilde{Y}}{E}\left[D_{y}\left(\tilde{y}\right)\right]-\underset{y∼Y}{E}\left[D_{y}\left(y\right)\right]\\ \; & \;+\lambda_{GP_{y}}\underset{\hat{y}∼\hat{Y}}{E}\left[{\left(∥\nabla_{\hat{y}}D_{y}\left(\hat{y}\right){∥}_{2}-1\right)}^{2}\right] \end{array}$$

where $\lambda_{GP_{y}}$ is a scalar hyperparameter defined as 10, $\hat{y}=\delta y+\left(1-\delta \right)\tilde{y}$ (given δ∼U[0,1]) and ∇ is the differential operator. The third term in Equation (7) is a regularization term that penalizes the gradient during training in order to achieve the constraint that discriminators must be 1-Lipschitz functions [19].

Finally, the objective of $L_{cyc}$, the second term of Equation (4), is

(8) $$\begin{array}{cc} L_{cyc}\left(G_{xy},G_{yx},X,Y\right) & =L_{cyc}^{x}\left(G_{xy},G_{yx},X\right)+L_{cyc}^{y}\left(G_{xy},G_{yx},Y\right)\\ \; & =\underset{x∼X}{E}\left[{∥G_{yx}\left(G_{xy}\left(x\right)\right)-x∥}_{1}\right]+\underset{y∼Y}{E}\left[{∥G_{xy}\left(G_{yx}\left(y\right)\right)-y∥}_{1}\right] \end{array}$$

#### 2.3.4. Hyperparameters and Experimental Settings

All experiments were performed in tge Google Collaboratory environment. The training and test set proportions were 85% and 15%, respectively, from the total virtual subjects mentioned in Section 2.1. The CycleGAN parameters were updated using the Adam optimizer [20], the learning rate (LR) value was $10^{-4}$, and the batch size was equal to 96. All the hyperparameters are shown in Table 1. In particular, the number of discriminators per generator updates ($D_{Iters}$) was only considered for the WGAN-GP case [21], while for the LSGAN case it was always 1. The normalization layers and last activation function for LSGAN were batch-normalization and sigmoid function, respectively, while for WGAN-GP they were instance-normalization and linear function, respectively. Models were trained for 1750 epochs.

### 2.4. Evaluation

After the training was finished, the only relevant part of the CycleGAN interesting for clinical purpose was $G_{xy}$. Recalling that the signals were within the MinMax ranges defined in Section 2.1, the predictions were returned to the original scale. Furthermore, evaluation was performed only across the first completed pulse presented in both the test and estimated signals. Two scopes were considered. The first was a typical ML metric, using the root-mean squared error (RMSE) for both pressure and area prediction:

(9) $$RMSE=\sqrt{\frac{1}{N}\sum_{i=1}^{N}\frac{1}{T}\sum_{j=1}^{T}{\left(z_{ij}-{\hat{z}}_{ij}\right)}^{2}}$$

where *N*, *T*, *z*, and $\hat{z}$ refer to the number of samples, pulse duration, ground truth pulse, and estimated pulse, respectively.

Second, in order to evaluate the elastic properties of the predictions, E_P-ε_ was calculated for both the ground truth and estimated pulses. The strain ε was derived from the area PW, then E_P-ε_ is computed as the slope β of a simple linear regression:

(10) $$\hat{\beta }=\sum_{j=1}^{T}\left(\varepsilon_{j}-\bar{\varepsilon }\right)\left(P_{j}-\bar{P}\right)/\sum_{j=1}^{T}{\left(\varepsilon_{j}-\bar{\varepsilon }\right)}^{2}$$

After calculating each $\beta_{i}$, mean error (ME) and mean absolute percentage error (MAPE) were evaluated regarding E_P-ε_ as follows:

(11) $$ME=\frac{1}{N}\sum_{i=1}^{N}\left(\beta_{i}-{\hat{\beta }}_{i}\right)$$

(12) $$MAPE=\frac{100\%}{N}\sum_{i=1}^{N}(\left|\beta_{i}-{\hat{\beta }}_{i}\right|/\beta_{i})$$

To account for the possible differences in duration between the estimated and true pulses, the shorter one was used. Finally, the minimum and maximum values for pressure and diameter were extracted to perform a Bland–Altman analysis.

## 3. Results

**Figure 5:**
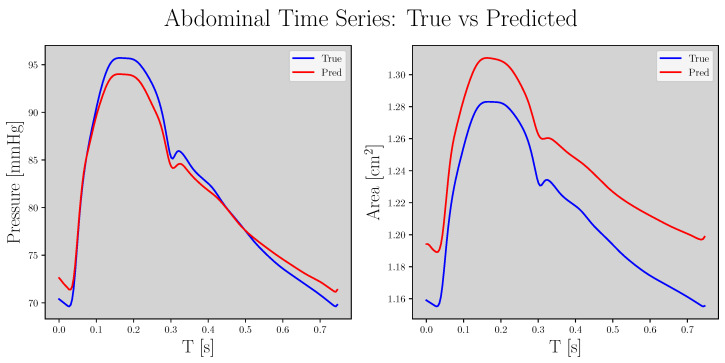
Comparison between true and predicted signals for pressure (**left**) and area (**right**); blue and red correspond to the true and predicted signals, respectively.

The statistical results for the test set are shown in Table 2 for the best hyperparameter combination of each $L_{GAN}$. The means ± standard deviation of error during evaluations for the best LSGAN and WGAN-GP were 0.8 ± 0.4 mmHg and 1.7 ± 0.8 mmHg, respectively. For area evaluations, the results were 0.1 ± 0.1 cm^2^ and 0.2 ± 0.2 cm^2^, respectively. Figure 5 compares the true and predicted samples.

**Figure 6:**
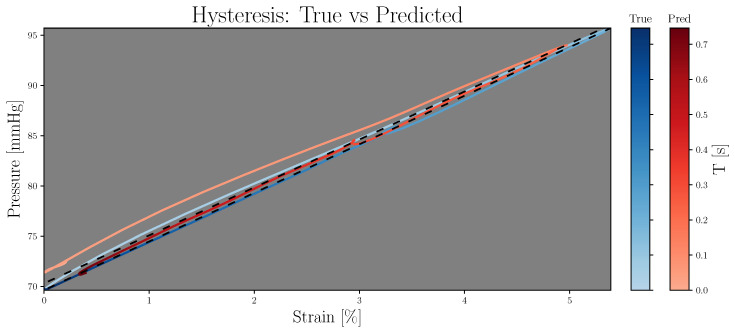
Comparison between true (blue) and predicted (red) hysteresis cycles. Line brightness refers to time; the black dashed lines refer to β and $\hat{\beta }$ from Equation (10).

Concerning E_P-ε_, the ME for the best LSGAN and WGAN-GP results were 13.1 ± 56.5 mmHg/% and 70.6 ± 216.0 mmHg/%, respectively. Finally, according to MAPE, the LSGAN and WGAN-GP results were 6.5 ± 5.1% and 28.6 ± 19.3%, respectively. Figure 6 shows a hysteresis comparison for the same signals in Figure 5, where the black dotted line represents the β and $\hat{\beta }$ parameters from Equation (10).

The esults in Table 3 corresponding to the training set are presented to demonstrate that the error does not suffer from overfitting.

**Figure 7:**
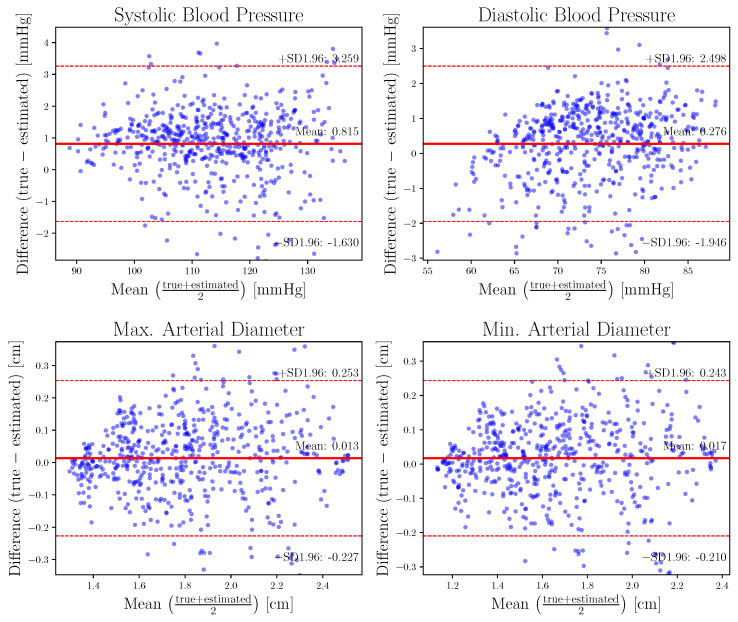
Bland–Altman plots showing the difference between the maximum and minimum values of pressure (**top**) and diameter (**bottom**) for true and estimated signals; the plots were obtained for the best LSGAN model.

In particular, the Bland–Altman plots (Figure 7) for the SBP and DBP values for the LSGAN best model (Table 2, experiment A) express a mean difference of 0.81 and 0.27 mmHg, respectively. It is worth repeating that the SBP and DBP values are the maximum and minumum values of the pressure signal, respectively. Limits of agreement (LOA) are [+3.25, −1.63] mmHg for SBP, while the LOA for DBP are [+2.49, −1.94] mmHg. Concerning the maximum and minimum values of the diameter, the Bland–Altman plots show a mean difference of 0.013 and 0.017 cm, respectively. limits of agreement LOA for the maximum diameter are [+0.253, −0.227] cm, while LOA for the minimum diameter are [+0.243, −0.210] cm.

## 4. Discussion

In this paper, a new methodology based on a deep learning technique (CycleGAN) is proposed to estimate pressure vs. strain dynamics. While the majority of the literature considers the estimation of PWV using the feature extraction approaches, in this paper two peripheral pressure PWs are considered to estimate central pressure and area PWs. Consequently, a wider approach to the aortic dynamics is achieved in terms of the P-D relationship. According to the literature [16,22], after these two PW are estimated both the ground truth and estimated hysteresis loop can be constructed and evaluated through assessment of each E_P-ε_. It is worth noting that the obtained P-D loops correspond to an aortic site where the real measurement would, while highly invasive, be very useful regarding the potential information that could extracted [23,24].

Regarding the LSGAN model, and considering that it was not particularly trained to estimate calibration values, visual inspection of the Bland–Altman plots in Figure 7 does not suggest any heteroscedasticity. In relation to systematic bias, DBP seems to be slightly overestimated as values increase. It is observed that estimation of pressure PW values shows a lower relative error compared to area estimation. It is hypothesized that calibration similarities between P_Bra-Rad_ and P_Abdo_ reduce the learning difficulty and offer bounds on the estimated PW. For example, systolic P_Bra-Rad_ values are always greater than P_Abdo_, and the model can easily learn this, while the shape of the PW remains as a challenging task. On the contrary, the calibration of the area PW values has no reference points from the pressure values, meaning that the learning task increases in complexity. In particular, for the pressure PW, in comparison with previous works [25,26,27] it is apparent that the error as presented in Table 2 is lower. It should be noted, however, that these previous works were obtained from real subjects, which is a more challenging task.

It is worth mentioning that a similar model was used in Brophy et al. [28], where domain transformation was performed from finger PPG to peripheral BP and signals were taken from subjects in intensive care units. In Brophy et al. [28], evaluation was performed only with respect to the mean BP and trained with a single $L_{GAN}$, while in the present approach two $L_{GAN}$ were explored, a generator $G_{xy}$ returned both pressure and area values, and the evaluation was performed across all the PWs and their inter-relationship (P-D loop).

Although the best hyperparameter results were obtained with LSGAN, it is noteworthy that WGAN-GP results are less sensitive to hyperparameter search and more stable in terms of training. Increasing the number of epochs could produce better results; however, considering our resource capacity, 1750 epochs seems to offer a fair comparison between $L_{GAN}$. In Appendix A, Table A1 and Table A2, the complete results of the grid search and their corresponding hyperparameters as proposed in Table 1 are reported. Finally, to ensure collaborate with the community and for ease of reproducibility, our code is publicly available (please see Supplementary Materials section)

In order to validate the proposed methodology and open new lines of research, in vivo P-D measurements such as those detailed in [29,30] should be performed. It is noteworthy that the virtual subjects considered in this study were simulated assuming a healthy condition. In addition, the impact of the surrounding tissue on the mechanical behavior of the arteries was not taken into account [31]. Lastly, as a future line of work, it would be interesting to evaluate model performance considering subjects with particular CV diseases.

## 5. Conclusions

The ability of GAN-based deep learning models to transfer pressure signals from the peripheral artery regions to pressure and area signals in the central arterial region is analyzed in this paper. Unlike previous studies that used PWV to quantify arterial stiffness, the present study reconstructs and evaluates the pressure–strain hysteresis loop. When adversarial losses from LSGAN and WGAN-GP are compared, LSGAN provides more accurate results.

## Figures and Tables

**Table 1:** Grid search of hyperparameters. $L_{GAN}$ refers to the adversarial loss function. $G_{GRU}$ and $D_{l=1}$ refer to the feature size of the generator’s GRU units and the first layer output’s discriminators. $D_{Iters}$ refers to the number of discriminators per generator updates (only for WGAN-GP; for LSGAN it is always 1).

$L_{\mathrm{GAN}}$	$G_{\mathrm{GRU}}$	$D_{l=1}$	$D_{\mathrm{Iters}}$	$\lambda_{\mathrm{cyc}}$
[LSGAN, WGAN-GP]	[64, 128]	[6, 8]	[5, 15, 25]	[5, 15, 25]

**Table 2:** Mean ± standard deviation of error for Brachial–Radial to Aortic Abdominal case on the test set.

$L_{\mathrm{GAN}}$	Experiment	Pressure [mmHg]	Area [cm^2^]	$E_{\mathrm{P-\varepsilon}}$ [mmHg/%]	
		RMSE	RMSE	ME	MAPE
LSGAN	A	0.8 ± 0.4	0.1 ± 0.1	13.1 ± 56.5	6.5 ± 5.1
WGAN-GP	B	1.7 ± 0.8	0.2 ± 0.2	70.6 ± 216.0	28.6 ± 19.3

A→ *G_GRU_* = 128, *D*_*t*=1_ = 8, *D_Iters_* = 1, *λ_cyc_* = 5. B→ *G_GRU_* = 64, *D*_*t*=1_ = 6, *D_Iters_* = 15, *λ_cyc_* = 25.

**Table 3:** Mean ± standard deviation of error for Brachial–Radial to Aortic Abdominal case on training set.

$L_{\mathrm{GAN}}$	Experiment	Pressure [mmHg]	Area [cm^2^]	$E_{\mathrm{P-\varepsilon}}$ [mmHg/%]	
		RMSE	RMSE	ME	MAPE
LSGAN	A	0.8 ± 0.4	0.1 ± 0.1	13.4 ± 51.5	6.2 ± 4.9
WGAN-GP	B	1.8 ± 0.9	0.2 ± 0.2	71.1 ± 209.3	28.3 ± 20.8

A→ *G_GRU_* = 128, *D*_*t*=1_ = 8, *D_Iters_* = 1, *λ_cyc_* = 5. B→ *G_GRU_* = 64, *D*_*t*=1_ = 6, *D_Iters_* = 15, *λ_cyc_* = 25.

## Data Availability

The database is publicly available at https://doi.org/10.5281/zenodo.3275625.

## Supplementary Materials

Source codes are available at https://github.com/AguirreNicolas/ABPCycleGAN.

## Abbreviations

The following abbreviations are used in this manuscript:

BP	blood pressure
CNN	convolutional neural network
CV	cardiovascular
CVD	cardiovascular disease
DBP	diastolic blood pressure
E	elastic module
E_P-ε_	pressure–strain elastic modulus
GAN	generative adversarial network
GRU	gated recurrent unit
LOA	limit of agreement
LR	learning rate
LSGAN	least-square GAN
MAPE	mean absolute percentage error
ME	mean error
ML	machine learning
NN	neural network
P-D	pressure–diameter
PPG	photoplethysmography
PW	pulse wave
PWV	pulse wave velocity
RMSE	root mean squared error
SBP	systolic blood pressure
SVM	support vector machine
WGAN-GP	Wasserstein GAN with gradient penalty

## Appendix A

**Table A1:** Experiments and their hyperparameters.

$L_{\mathrm{GAN}}$	$G_{\mathrm{GRU}}$	$D_{l=1}$	$D_{\mathrm{Iters}}$	$\lambda_{\mathrm{cyc}}$	Experiment
LSGAN	128	8	1	5	A
WGAN-GP	64	6	15	25	B
LSGAN	64	6	1	15	C
	64	6	1	25	D
	64	6	1	5	E
WGAN-GP	128	8	15	5	F
	64	6	10	5	G
	64	6	15	15	H
	64	6	15	5	I
	64	6	50	15	J
	64	6	50	25	K

**Table A2:** Detailed Brachial–Radial to Aortic Abdominal results.

$L_{\mathrm{GAN}}$	Experiment	Pressure [mmHg]	Area [cm^2^]	$E_{\mathrm{P-\varepsilon}}$ [mmHg/%]	
		RMSE	RMSE	ME	MAPE
LSGAN	A	0.8 ± 0.4	0.1 ± 0.1	13.1 ± 56.5	6.5 ± 5.1
WGAN-GP	B	1.7 ± 0.8	0.2 ± 0.2	70.6 ± 216.0	28.6 ± 19.3
LSGAN	C	4.4 ± 1.2	0.2 ± 0.1	229.3 ± 304.6	45.0 ± 25.7
	D	27.1 ± 6.3	0.2 ± 0.1	654.2 ± 265.4	137.5 ± 14.6
	E	5.8 ± 3.3	0.3 ± 0.2	18.0 ± 137.7	17.3 ± 13.1
WGAN-GP	F	2.7 ± 1.7	0.4 ± 0.2	3.1 ± 349.1	59.7 ± 54.4
	G	3.8 ± 1.8	0.2 ± 0.1	84.3 ± 201.0	28.7 ± 19.5
	H	1.5 ± 0.7	0.4 ± 0.3	104.8 ± 222.3	28.7 ± 22.7
	I	3.4 ± 2.1	0.3 ± 0.2	10.9 ± 273.3	39.3 ± 31.9
	J	1.6 ± 0.7	0.4 ± 0.2	12.7 ± 300.1	48.2 ± 47.0
	K	2.0 ± 0.8	0.2 ± 0.2	68.4 ± 242.7	32.9 ± 23.6
